# Locating and Treating Three Calcified Canals in a Mandibular First Molar With Radix Entomolaris and Five Canals: A Case Report

**DOI:** 10.7759/cureus.52931

**Published:** 2024-01-25

**Authors:** He Liu, Ya Shen

**Affiliations:** 1 Division of Endodontics, Department of Oral Biological & Medical Sciences, University of British Columbia, Vancouver, CAN; 2 Division of Endodontics, Department of Oral Biological & Medical Sciences, University of British Columbia, Vacnouver, CAN

**Keywords:** radix entomolaris, root canal calcification, root canal treatment, middle mesial canal, permanent mandibular first molar, endodontic treatment

## Abstract

The success of root canal treatment is highly contingent on the comprehensive shaping, cleaning, and filling of the entire root canal system. Failure to address one or more canals often results in an increased likelihood of post-treatment apical periodontitis. Typically, mandibular first molars feature two roots and three canals, but they may also exhibit anatomical variations, such as a mesial middle canal or radix entomolaris (RE). This article presents a case where three calcified canals in a mandibular first molar with RE and five canals were successfully located and treated.

## Introduction

Apical periodontitis (AP) is an inflammatory response of periapical tissues primarily instigated by bacterial biofilm within the root canal system [1]. This biofilm, a structured bacterial cell community encased in a matrix of extracellular polymeric substances, triggers an immune response due to pathogen presence and their toxins [2-4]. The primary treatment for AP is root canal therapy, which focuses on eradicating bacterial infections, preventing reinfection, and fostering the healing of affected periapical tissues. The effectiveness of root canal treatment hinges on meticulously cleaning and shaping the root canals to remove diseased tissue, bacteria, and their byproducts [5-11], followed by filling the canals with an inert material to preclude future microbial ingress [12-16].

Mandibular first molars, the first permanent teeth to emerge in the oral cavity [17], are more prone to bacterial infection, dental caries, pulp tissue inflammation, necrosis, and AP due to their functional and morphological characteristics, as well as surrounding oral conditions [18]. The complexity of their root anatomy and intricate canal system poses significant challenges in effective cleaning and shaping during root canal procedures [17,18]. Typically, these molars have two roots (mesial and distal) and three canals [17,18], with the mesial root often housing two canals (mesial buccal [MB] and mesial lingual [ML]) and the distal root containing one or two canals [17,18]. The occurrence of a third root in mandibular first molars was found to be 13% [18]. In these molars, three canals were present in 61.3% of cases, four canals in 35.7%, and five canals in about 1% [18]. Instances of mandibular first molars with six, and even seven canals, have also been reported [18]. A common variation is the mesial middle (MM) canal located between the MB and ML canals in the mesial root [19]. A meta-analysis examining the global prevalence of MM canals in mandibular first molars via cone-beam computed tomography (CBCT) scans included 33 studies with 13,349 molars, revealing a global prevalence of 4.4% [19].

Another variant, radix entomolaris (RE), is an additional distolingual root, typically shorter and more curved than the distobuccal root (DB) [20]. A multinational cross-sectional study with meta-analysis using CBCT images to assess the prevalence of RE in mandibular first molars evaluated 6,304 CBCTs, representing 12,608 molars. The prevalence of RE varied from 0% to 12%, with an overall prevalence of 3% [20]. The association between missed canals due to anatomical variations and post-treatment AP is significant, with incomplete bacterial biofilm removal from the root canal system potentially leading to AP onset or persistence [21,22]. Therefore, understanding the anatomical variations of mandibular first molars is crucial for successful root canal procedures.

Another challenge to root canal treatment success is pulp chamber obliteration, particularly in locating and treating all root canals [22,23]. Sener et al. studied pulp chamber calcifications’ prevalence through radiographic examinations of 15,326 teeth from 536 patients, finding calcifications in 38% of patients and 4.8% of teeth examined [23]. There was a notable correlation between pulp chamber calcifications and gender, dental status, and higher prevalence in carious, restored, and restored plus carious teeth.

This case report presents a successful root canal treatment of a mandibular first molar with RE and five canals. Utilizing a dental operating microscope (DOM) and ultrasonic instruments, three calcified canals (ML, MM, and DB) were identified and treated. At the two-year follow-up, the tooth remained asymptomatic.

## Case presentation

A 22-year-old Chinese female patient was referred to the department of endodontics from a local dental clinic for treatment of the lower right first molar. During a pre-pregnancy oral health check, extensive carious lesions and a periapical lesion were discovered in tooth #46, which was asymptomatic. Root canal treatment was recommended due to the extensively compromised tooth structure, where only two canal orifices were identifiable owing to severe calcification. The dentist who began the root canal treatment suspected one or two unlocated canals and temporarily filled the tooth with zinc oxide-eugenol (ZOE). At the time of consultation, tooth #46 remained asymptomatic. The patient’s medical history was unremarkable, and her overall health was good (ASA I classification), with no systemic disease symptoms. She maintained excellent oral hygiene and had no harmful or parafunctional habits.

**Figure 1:**
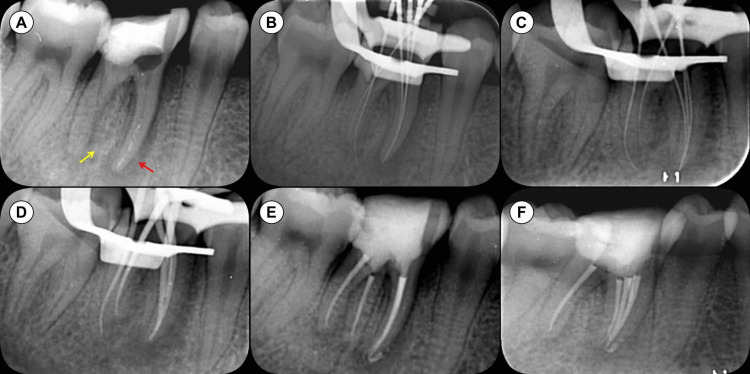
Periapical radiographs taken during root canal procedures for tooth #46 (A) The pre-operative radiograph of tooth #46 reveals previous treatment and significant structural reduction, along with a “J” shaped periapical lesion around the mesial root (indicated by a red arrow). There is radiopaque material in the mesial canals. Additionally, the tooth features an extra distolingual root known as radix entomolaris (yellow arrow). (B,C) Periapical radiographs captured from two different angles to ascertain the working length. (D) A periapical radiograph during the fitting of the master gutta-percha cones. (E,F) Post-obturation periapical radiographs, each taken from a different angle.

Clinical examination of tooth #46 revealed normal gingiva, no palpation tenderness at the root apex, normal tooth mobility, and a negative percussion test. Periodontal probing was within normal limits. A periapical radiograph showed extensive damage to the coronal tooth structure and pulp chamber floor (Figure 1A), a “J” shaped periapical lesion around the mesial root, and radiopaque material in the mesial canals. Based on these findings, tooth #46 was diagnosed with previously initiated and asymptomatic AP. The treatment plan included root canal treatment followed by full crown restoration. The patient was informed about and consented to the treatment plan and procedures.

**Figure 2:**
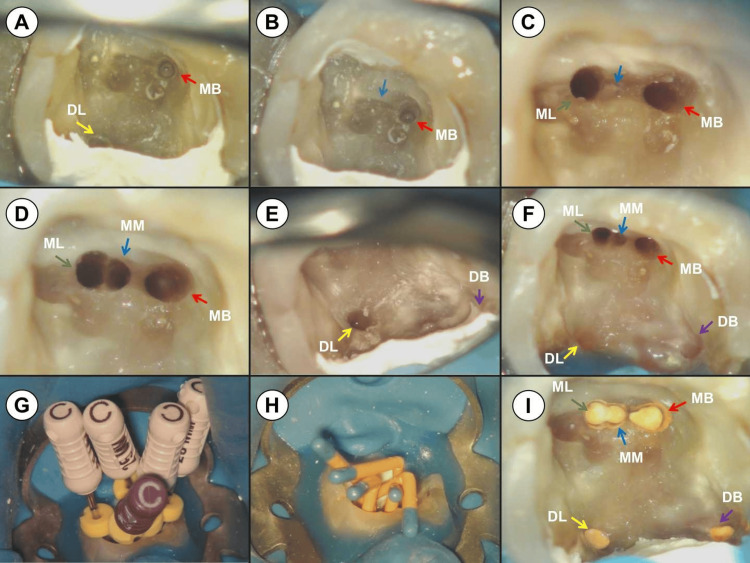
Photographs of root canal procedures for tooth #46 taken under a dental operating microscope (A) The pre-operative photograph reveals extensive damage to the pulp chamber floor from previous treatment. The orifices of the mesial buccal canal (MB, indicated by a red arrow) and the distal lingual canal (DL, indicated by a yellow arrow) are visible. (B) A dark line (marked by a blue arrow) suggests a potential developmental groove linking the MB canal orifice. (C) The photograph displays the developmental groove connecting the MB canal orifice (red arrow) to the mesial lingual canal orifice (ML, green arrow). Near the ML canal orifice, a white spot (blue arrow) filled with dentin debris is evident. (D) The photograph illustrates the developmental groove linking the MB canal orifice (red arrow), the distal buccal canal orifice (DB, green arrow), and the mesial middle canal orifice (MM, blue arrow). (E) The DL canal orifice (yellow arrow) and the potential location of the distal buccal canal orifice (DB, purple arrow) are shown. (F) The photograph displays the MB canal orifice (red arrow), ML canal orifice (green arrow), MM canal orifice (blue arrow), DB canal orifice (purple arrow), and DL canal orifice (yellow arrow). (G) The photograph demonstrates the use of C-Pilot files for canal negotiation and working length determination. (H) The photograph shows #30/.04 gutta-percha cones being used for master cone fitting. (I) A post-obturation photograph reveals that the MB canal (red arrow), ML canal (green arrow), MM canal (blue arrow), DB canal (purple arrow), and DL canal (yellow arrow) have been filled with gutta-percha and bioceramic sealer.

After rubber dam isolation, the temporary filling was removed, and access was gained under DOM (OPMI PICO, Carl Zeiss, Oberkochen, Germany). The orifices of the MB and distal lingual (DL) canals were visible under the DOM (Figure 2A). Extensive damage to the pulp chamber floor was noted, as a result of efforts to locate the ML canal orifice in previous treatments. A dark line suggested a developmental groove connecting the MB and ML canal orifices (Figure 2B). Following the “law of orifice location 3,” a diamond ultrasonic tip (ET18D; Satelec Acteon Group, Merignac, France) was used to trace the dark line, uncovering the ML canal orifice after further dentin removal with a stainless steel ultrasonic tip (ET21; Satelec Acteon Group) (Figure 2C) [24]. A white spot near the ML canal orifice, filled with dentin debris, indicated the potential presence of an MM canal orifice. This was exposed using the ET21 ultrasonic tip (Figure 2D). Following the “laws of symmetry 2,” the ET18D and ET21 tips were employed to locate the DB canal orifice (Figure 2E), which was successfully identified [24]. All five canals of tooth #46 were located with the aid of the DOM and ultrasonic instruments (Figure 2F). C-Pilot files (VDW, Munich, Germany) were used to negotiate the canals, the working lengths were measured with an electronic apex locator (J Morita Corp, Tokyo, Japan), and periapical radiographs from different angles confirmed these lengths (Figures 1B, 1C, 2G). The canals were shaped to #30/.04 using the Twisted Files NiTi rotary system (TF; SybronEndo, Orange, CA, USA), with alternating irrigation of 3% sodium hypochlorite (NaOCl) and 17% ethylenediaminetetraacetic acid (EDTA). The canals were then thoroughly rinsed with sterile water and dried with paper points. Calcium hydroxide paste (Pulpdent™ paste; Pulpdent Corporation, Watertown, MA, USA) was introduced, and the access cavity was temporarily sealed.

One week later, the patient returned. The temporary filling was removed, and the canals were rinsed with 3% NaOCl and sterile water. The Irri-Safe ultrasonic tip (Satelec Acteon Group) removed the calcium hydroxide, followed by trial fitting of the main gutta-percha cones (Figures 1D, 2H). After drying the canals with paper points, the iRoot SP bioceramic sealer (Innovative Bioceramix, Vancouver, Canada) was injected into the upper portion of the canals. The gutta-percha cones, dipped in a small amount of iRoot SP sealer, were placed in the canals, and the root canal filling was completed using the Elements Obturation Unit (SybronEndo). The gutta-percha cones were cut at canal orifice level using a heat carrier tip (SybronEndo), and a Buchanan hand plugger (SybronEndo) compacted the heated gutta-percha (Figure 2I). Two periapical radiographs from different angles evaluated the root canal filling quality (Figures 1E, 1F), and the access cavity was again temporarily sealed. The patient was advised to wait two weeks before proceeding with the full crown restoration.

At a three-month follow-up phone call, the patient, who had refused to come in for examinations, reported that tooth #46 had been fully crowned, was asymptomatic, and functioning well. Two years later, another phone call for a follow-up revealed that she had recently given birth and was breastfeeding. She declined in-person follow-up examinations but reported that the tooth remained asymptomatic and functional.

## Discussion

The anatomical complexities of roots and root canal systems in mandibular first molars pose considerable challenges for root canal procedures, especially in locating, cleaning, shaping, disinfecting, and filling the root canals [17-20]. These challenges are further exacerbated by severe calcification within the pulp chamber. Failing to locate, clean, and shape all root canals adequately can result in root canal treatment failure [22,23]. There is a well-established connection between missed canals and the development of post-treatment AP, primarily due to the incomplete eradication of bacterial biofilm from the root canal system, which can lead to the onset or persistence of AP [22,23]. In our case, the initial dentist who performed the root canal treatment on the mandibular first molar identified only two canals (MB and DL). Typically, mandibular first molars have three canals, indicating at least one canal was missed. Moreover, according to the “law of symmetry 2,” which states that for teeth other than maxillary molars, the canal orifices are aligned along a line perpendicular to a mesial-distal line across the center of the pulp chamber floor [24], it was deduced that there should be ML and DB canals in our case. This law implies that the line connecting the MB and ML canal orifices and the line connecting the DB and DL canal orifices should be perpendicular to a mesial-distal line running through the pulp chamber floor. These observations led us to suspect the presence of at least two missed canals in this patient.

To effectively locate all roots and root canals, clinicians should have a deep understanding of the anatomical variations of the teeth being treated and obtain high-quality periapical radiographs from various angles during the pre-operative diagnosis [25]. This approach aids in detecting additional roots or root canals [25]. Wang et al. conducted an in vitro study evaluating the optimal X-ray projection angle for diagnosing RE in mandibular first molars [25]. In this study, 25 extracted mandibular first permanent molars with RE were subjected to radiographic examination at seven different horizontal angles. The study concluded that radiographs taken at an additional 25° mesial horizontal angle are most effective for preoperative identification and assessment of RE.

When needed, limited field-of-view CBCT images can offer three-dimensional localization of the root canal orifice, enabling measurements of the distance between blocked orifices and reference points, as well as the thickness of any obstructions [26-28]. This method facilitates more precise procedures, minimizing damage to the tooth structure and avoiding perforation of the pulp chamber floor or root canal. However, access to CBCT imaging might be limited for patients with financial constraints or those who decline CBCT scans [26-28]. Nonetheless, adherence to the “as low as reasonably achievable (ALARA)” principle is crucial to ensure patient safety [26-28].

Understanding pulp chamber anatomy and adhering to anatomic laws regarding the pulp chamber floor can assist in determining the pulp chamber’s position, as well as the precise location and number of root canals in a given tooth [24]. In a classic anatomical study, Krasner and Rankow proposed several anatomic laws after examining 500 extracted teeth’s pulp chambers [24]. They noted that developmental root fusion lines are darker than the floor and that root canal orifices are situated at the end of these lines (law of orifice location 3) [24]. In our case, this law aided in locating the ML and MM canals. Upon removal of the temporary filling material, a dark line in the mesial part of the pulp chamber suggested a potential developmental root fusion line. Following this anatomic law, the ML canal orifice was anticipated at the lingual end of the dark line. Consequently, ultrasonic tips were employed to remove calcification along this line, revealing the ML canal precisely at the expected location.

Another applied principle was the “law of symmetry 2”: for teeth other than maxillary molars, the canal orifices align on a line perpendicular to a mesial-distal line across the pulp chamber floor center [24]. In our case, this meant that the line connecting the DL and DB canal orifices should be perpendicular to a mesial-distal line through the pulp chamber floor. However, the DL and DB canal orifices were not equidistant from a mesial-distal line, not fully adhering to the “law of symmetry 1” [24]. This deviation highlights that while these anatomic laws serve as excellent guides, they are not infallible.

Illumination and magnification are critical for successfully identifying additional canals. Karapinar-Kazandag et al. studied the detection and negotiation of MM canals in mandibular molars using magnifying loupes and an operating microscope [29]. With magnifying loupes, eight (16%) MM canals were detected, and six (12%) negotiated in mandibular first molars. The numbers increased to nine (18%) detected and seven (14%) negotiated with the use of DOM. Troughing the pulp chamber floor with ultrasonic instruments or long shank round burs, facilitated by DOM, is recommended for locating additional root canals [30,31]. This procedure entails minimal dentin removal between the MB and ML canals, moving mesio-apically away from the furcal danger zone [30,31].

Azim et al. conducted an in vivo study to determine the prevalence of MM canals before and after troughing using a standardized technique [30]. In 91 mandibular molars from 87 patients, six MM canals were located after conventional access (6.6%), with an additional 36 found post-troughing (39.6%), totaling 42 MM canals (46.2%). They concluded that troughing up to a 2-mm depth in the mesial root is an effective and safe method to identify potential MM canals, with deeper troughing possibly leading to root perforation.

In a micro-computed tomographic study, Keleş and Keskin assessed the depth of MM canal orifices and their detectability in mandibular molars using troughing preparation [31]. Their findings indicated that 77.41% of MM canal orifices were at the cementoenamel junction (CEJ) level, with 5.38% and 9.69% detectable within 1-mm and 2-mm depths from the CEJ, respectively. However, 7.52% of the orifices were deeper than 2 mm from the CEJ. They concluded that while 77.41% of mandibular molars might not require troughing, 15.07% would benefit from it, and 7.52% could not be accessed even with troughing preparation. In this case report, utilizing a DOM, a diamond, and a stainless steel ultrasonic tip, a 1 mm deep trough was created along the potential developmental groove. The ML canal was first exposed, followed by the identification of a white spot filled with dentin debris near the ML canal orifice, suggesting an MM canal. This was uncovered using the ET21 ultrasonic tip. This case exemplifies the effectiveness of the troughing technique with DOM and ultrasonic instruments in locating calcified canals.

## Conclusions

For successful identification of additional roots or root canals, clinicians must have a thorough understanding of the anatomical variations in mandibular first molars. Periapical radiographs, taken from various angles, can aid in the preoperative diagnosis of these additional roots or canals. Employing anatomic laws as a guide, troughing the pulp chamber floor using ultrasonic instruments, especially when facilitated by a DOM, has proven effective in locating calcified canals in mandibular first molars.
